# Context- and Cell-Dependent Effects of Delta-Like 4 Targeting in the Bone Marrow Microenvironment

**DOI:** 10.1371/journal.pone.0052450

**Published:** 2012-12-20

**Authors:** Leonor Remédio, Tânia Carvalho, Francisco Caiado, Ana Bastos-Carvalho, Diana Martins, António Duarte, Hideo Yagita, Sergio Dias

**Affiliations:** 1 Angiogenesis Lab, CIPM, Portuguese Institute of Oncology, IPOLFG, EPE, Lisbon, Portugal; 2 Department of Immunology, Juntendo University, School of Medicine, Tokyo, Japan; 3 CIISA, Faculdade de Medicina Veterinária, Technical University of Lisbon, Lisbon, Portugal; 4 CEDOC, FCM, Universidade Nova de Lisboa, Lisbon, Portugal; 5 Instituto Gulbenkian de Ciência, Oeiras, Portugal; 6 Programme for Advanced Medical Education, Centro Hospitalar Lisboa Norte and Faculdade de Medicina de Lisboa, Lisbon, Portugal; 7 Department of Ophthalmology, Centro Hospitalar Lisboa Norte and Faculdade de Medicina de Lisboa, Lisbon, Portugal; 8 Obstetrics and Gynecology Department, Hospital de Santa Maria, Lisbon, Portugal; Katholieke Universiteit Leuven, Belgium

## Abstract

Delta-like 4 (Dll4) is a ligand of the Notch pathway family which has been widely studied in the context of tumor angiogenesis, its blockade shown to result in non-productive angiogenesis and halted tumor growth. As Dll4 inhibitors enter the clinic, there is an emerging need to understand their side effects, namely the systemic consequences of Dll4:Notch blockade in tissues other than tumors. The present study focused on the effects of systemic anti-Dll4 targeting in the bone marrow (BM) microenvironment. Here we show that Dll4 blockade with monoclonal antibodies perturbs the BM vascular niche of sub-lethally irradiated mice, resulting in increased CD31^+^, VE-Cadherin^+^ and c-kit^+^ vessel density, and also increased megakaryocytes, whereas CD105^+^, VEGFR3^+^, SMA^+^ and lectin^+^ vessel density remained unaltered. We investigated also the expression of angiocrine genes upon Dll4 treatment *in vivo*, and demonstrate that IGFbp2, IGFbp3, Angpt2, Dll4, DHH and VEGF-A are upregulated, while FGF1 and CSF2 are reduced. In *vitro* treatment of endothelial cells with anti-Dll4 reduced Akt phosphorylation while maintaining similar levels of Erk 1/2 phosphorylation. Besides its effects in the BM vascular niche, anti-Dll4 treatment perturbed hematopoiesis, as evidenced by increased myeloid (CD11b^+^), decreased B (B220^+^) and T (CD3^+^) lymphoid BM content of treated mice, with a corresponding increase in myeloid circulating cells. Moreover, anti-Dll4 treatment also increased the number of CFU-M and -G colonies in methylcellulose assays, independently of Notch1. Finally, anti-Dll4 treatment of donor BM improved the hematopoietic recovery of lethally irradiated recipients in a transplant setting. Together, our data reveals the hematopoietic (BM) effects of systemic anti-Dll4 treatment result from qualitative vascular changes and also direct hematopoietic cell modulation, which may be favorable in a transplant setting.

## Introduction

Hematopoiesis is the process by which new blood cells are generated and occurs mainly in the adult bone marrow (BM). The importance of the BM microenvironment in regulating hematopoiesis has been amply demonstrated by studying the so-called “stem cell niches”, in which the endosteal and vascular niches were shown to support hematopoietic stem cells (HSCs) self-renewal, proliferation, and differentiation [1]–[4]. However, recent findings have proven this interpretation of the BM stem cell niches may to be too simplistic [5], [6]. Interestingly, the vascular niche is not only critical for HSC maintenance[7]–[9] and differentiation [10], but also for hematopoietic reconstitution and recovery [11]–[15]. Mechanistically, the BM endothelial cells were shown to express different “angiocrine genes”, whose production is dependent on the activation of Akt or MAP kinase signaling pathways [29], and whose function is to restore hematopoiesis following insults such as irradiation. Therefore, targeting the BM vascular niche and angiocrine genes production to modulate hematopoietic recovery and function may be of clinical relevance. We found Delta-like 4 (Dll4, a ligand of the Notch signaling pathway expressed by BM endothelial cells) targeting to potentially fulfill this aim.

Blockade of Dll4-mediated Notch signaling has been described as a modulator of tumor angiogenesis. Indeed, its inhibition, by promoting non-productive angiogenesis, was shown to be an effective treatment strategy in pre-clinical solid tumor models [16]–[19], and is already being tested in clinical trials [20], [21].

We have explored the effects of Dll4 blockade in the BM vascular niche using two strategies, first by using different endothelial cell markers, to assess qualitative changes in BM vasculature, and secondly by exploring the modulation of “angiocrine genes” *in vivo* and EC-specific activation of signaling pathways *in* *vitro*. To characterize the phenotypic response of the BM vascular niche to anti-Dll4 antibody treatment, we used different EC markers (CD31, CD105, VE-Cadherin, vascular endothelial growth factor receptor 3 (VEGFR3) and *Lycopersicon esculentum* lectin [22]–[24]), SMA (smooth muscle actin, a pericyte marker) [25], and by counting megakaryocyte numbers (which are part of the BM vascular niche, and are CD41^+^ [26]–[28]). Additionally, we assessed the effect of Dll4 blockade in modulating the expression of angiocrine genes [29] and activation of signaling pathways on BM endothelial cells *in vitro*.

We also determined how Dll4 systemic blockade interfered with hematopoiesis by directly affecting hematopoietic cells. Dll4 has been shown to be involved in HSCs self-renewal and proliferation [30]–[32], megakaryocytic differentiation [33], [34] and lymphoid modulation [33], [35]–[37]. However, the hematopoietic effects of Dll4 blockade, namely in the setting of perturbed BM function, had not been previously shown.

We performed *in vivo* phenotypic characterization of the main BM hematopoietic lineages following anti-Dll4 treatment, *in vitro* functional assays to identify hematopoietic cell-specific modulation of anti-Dll4, and an *in vivo* BM transplant (BMT) following lethal irradiation. For the *in vivo* characterization of the main BM hematopoietic lineages we quantified myeloid (CD11b^+^) and lymphoid (B, B220^+^ and T, CD3^+^) BM content [38]–[41]. Additionally, we measured hematopoietic stem/progenitor cells (HSPCs; stem cell antigen (Sca)-1^+^ and fetal liver kinase (Flk)-1^−^) [42], [43] and endothelial progenitor cells (EPC; Sca1^+^Flk1^+^ [44]–[46], in BM and peripheral blood (PB). The effects of anti-Dll4 treatment in HSPCs commitment and differentiation was assessed *in vitro* by performing colony-forming units (CFU) assays in methylcellulose [47], [48].

We show that systemic Dll4 blockade affects the BM vascular niche and hematopoietic cell differentiation, while having limited effects on the expression of “angiocrine genes” or on EC activation. Interestingly, in a BMT setting, anti-Dll4 treatment of donor mice results in faster lymphoid and erythroid recovery of recipient mice.

Together, we show that anti-Dll4 treatment perturbs BM recovery following irradiation, which can be clinically relevant in a BMT setting.

## Methods

### Animals and Experimental Design

The following animal experiments were performed following approval of the Instituto Gulbenkian de Ciência Animal Care Committee and Review Board.

Balb/c mice (6–8 weeks old) were sub-lethally irradiated (300rad), and subjected to treatment with neutralizing anti-mouse Dll4 antibody (HMD4-2) [19], [49], [50], 12.5 g/kg, intraperitoneally (IP), every 2 days or every 3 days, for 15 days, starting 1 day after irradiation. In parallel, control mice were injected with phosphate-buffered saline (PBS). All experiments refer to 15–20 days counting from the day of irradiation. Each irradiated group consisted of 3 control and 3 anti-Dll4 treated animals, and the experiments were performed 3 times.

The Dll4 knockout mice experiments were performed with the approval of the Faculty of Veterinary Medicine of Lisbon Ethics and Animal Welfare Committee. Dll4 conditional knockout mice (Dll4^lox/lox^) were generated as follows: conditional KO Dll4 vector with 2 loxP sequences flanking the 3 first gene exons was inserted in EE cells by electroporation. The neomycin resistant clones were selected, injected in blastocysts and transferred to pseudo-pregnant females. The offspring were crossed with h-ActB-flp mice to remove neoR, and the resulting littermates were crossed to obtain Flp^−/−^. These mice were then crossed with VECad^CreERT2^ mice, a gift from Dr. Ralph Adams, to produce a tamoxifen-inducible endothelial-specific Dll4 loss-of-function line (VECad^CreERT2^Dll4^lox/lox^). Tamoxifen induction was performed for 5 days, 50mg/kg/day. All experiments refer to 31–34 days counting from the first day of induction. Each group consisted of 12 Dll4^lox/lox^ and 11 VECad^CreERT2^Dll4^lox/lox^ animals.

### Sample Collection

Peripheral blood was collected from the heart in EDTA-coated tubes (Multivette 600, Sarstedt, Nümbrecht, Germany) and centrifuged at 1200 rpm for 5 minutes.

BM was flushed from the long bones with PBS 0.5% BSA and centrifuged at 800 rpm for 15 minutes. PB and BM cells were collected for FACS analysis.

Femur BM was flushed with PBS and immediately centrifuged at 800 rpm for 15 minutes. Plasma was then collected for enzyme-linked immunosorbent assay (ELISA) analysis.

### Bone Marrow Transplants

Balb/c mice (6 weeks old) were lethally irradiated (800rad), and subjected to BMT 24 hours later. Cells for BMT were collected from the femur of previously treated or control animals (two recipients per donor animal), on day 15 of treatment. Viable nucleated cells were counted in a Countless Automated Cell Counter (Invitrogen, Carlsbad, CA). 2.5×10^6^ total BM cells were injected intravenously. BM for BMT was collected from 3 control and 3 anti-Dll4 treated animals. Recipient animals were treated with enrofloxacin 10 mg/kg every day for 7 days post-irradiation.

Complete blood counts (CBC) of tail vein PB was performed at weeks 1 and 2 post-transplantation.

### Cell Culture

Human umbilical cord vein ECs (HUVECs) (Clonetics, Lonza, Switzerland) were cultured in EBM-2 supplemented with EGM-2 SingleQuots, 2 mg/mL BBE (Lonza, Walkersville, MD) and 10% heat-inactivated fetal bovine serum (FBS) (Gibco Invitrogen, Carlsbad, CA).

Murine bone marrow-derived stromal cell line S17 was cultured in complete medium – Roswell Park Memorial Institute (RPMI) 1640 medium, 2 mM L-Glutamine, antibiotic-antimycotic (all from Gibco Invitrogen, Carlsbad, CA) and 50 µM β-mercaptoethanol (Sigma-Aldrich, Germany) – plus 10% FBS.

### *In vitro* Colony Forming Assays

BM mononuclear Lin^−^Sca1^+^ cells (10^4^), collected from anti-Dll4 treated and control animals and sorted in FacsAria (Becton Dickinson, Franklin Lakes, NJ), were plated onto cytokine-supplemented methylcellulose medium (MethoCult GF M3434, Stem Cell Technologies, Vancouver, BC, Canada). Resulting colonies are single-cell derived and represent the original cell’s identity [47], [48]. Colonies were scored after 1 and 2 weeks of culture, according to the manufacturer’s instructions.

Human cord blood mononuclear cells were lineage depleted using lineage cell depletion kit, as shown in Table S1. 10^4^ Lin^−^ cells were plated onto cytokine-supplemented methylcellulose medium (MethoCult GF H4434, Stem Cell Technologies, Vancouver, BC, Canada). Treatment with neutralizing anti-human Dll4 antibody (MHD4-46) [51], [52], 50 µg/mL, and/or anti-human Notch1 antibody (MHN1-128) [53], 10 µg/mL, started the day after the establishment of the culture and was performed every 2 days. Colonies were scored after 1 week of culture, according to the manufacturer’s instructions.

### Flow Cytometry

BM and PB mononuclear cells were stained for T, B, myeloid and progenitor cell markers, using the antibodies indicated on Table S1, 1 h at 4°C. BM cells were stained for megakaryocytes, following the same protocol. Flow cytometry was performed on FACSCalibur and analyzed with Cell Quest Software (Becton Dickinson, Franklin Lakes, NJ).

### Histological and Immunohistochemical Analysis

Livers were formalin-fixed and processed for routine histopathology and immunohistochemistry. Bones were formalin-fixed, EDTA-decalcified and processed for routine histopathology. Immunohistochemistry for the antigens indicated on Table S1 was performed in the humerus, on 3 µm slices, at 3 distinct levels for each bone/mouse (40 µm distance). Sections were incubated with primary antibody at room temperature for 1 h, immunostaining proceeded according to the visualization system manufacturer’s instructions and counterstained with Mayer’s hematoxylin.

Immunofluorescence for the antigens indicated on Table S1 was performed in the humerus, on 3 µm slices. Primary antibodies were incubated at room temperature for 1 hour, secondary antibodies were incubated at room temperature for 2 hours. Slides were mounted with Vectashield mounting medium with DAPI (VectorLaboratories, Burlingame, CA).

### Vascular Perfusion

*Fluorescein isothiocyanate (FITC) Lycopersicon esculentum* lectin (VectorLaboratories, Burlingame, CA) was injected in the tail vein (100 µg, from a 500 µg/mL solution). Mice were euthanized 5 minutes later, and perfused with 4% paraformaldehyde (PFA) in PBS. Femur BM was then flushed off and further fixed in 4% PFA overnight, dehydrated in a sucrose gradient for one day, and cryopreserved in Tissue-Tek Optimum Cutting Temperature (Sakura, Torrance, CA).

Cryosections (15 µm) were stained with ToPro-3 (Table S1) plus 100 µg/mL ribonuclease A (Sigma-Aldrich, Germany) at 4°C overnight, to visualize nuclei, and mounted in Mowiol 4–88 (pH 8.5 in Tris-HCl and glycerol; Calbiochem Merk Millipore, Darmstadt, Germany).

### Western Blotting

Third passage HUVEC at 70% confluence were cultured in EBM-2 plus 1% FBS for 17 hours, left untreated or treated with neutralizing anti-human Dll4 antibody (MHD4-46) [51], [52], 50 µg/mL, or PBS, for 2 hours. Cells were then lysed with RIPA buffer (20 mM Tris pH 7.5, 150 mM NaCl, 5 mM KCl, 5 mM MgCl, 1% Triton X-100, protease inhibitor cocktail and 1 mM sodium orthovanadate), and equal amounts of proteins were subjected to SDS–polyacrylamide gel electrophoresis with 12% Mini-Protean TGX precast gel (BioRad, US). Proteins were transferred onto nitrocellulose membrane (Hybond-C Extra, GE Healthcare Life Sciences, Roosendaal, Netherlands) and subjected to standard immunoblotting with the antibodies indicated on Table S1.

### Reverse Transcriptase PCR (RT–PCR)

For *in vivo* assessments, total BM from control or anti-Dll4 treated mice was flushed off in PBS, centrifuged 1200 rpm 5 min, and collected to TRIzol Reagent (Invitrogen, Carlsbad, CA). For *in vitro* assessments, third passage HUVEC at 70% confluence were starved with EBM-2 plus 1% FBS overnight, and treated with neutralizing anti-human Dll4 antibody (MHD4-46) [51], [52], 50 µg/mL, or PBS, for 16 hours, then collected to TRIzol Reagent (Invitrogen, Carlsbad, CA).

RNA was extracted according to the manufacturer’s instructions. cDNA was produced with SuperScript II (Invitrogen, Carlsbad, CA) by using random-sequence hexamer primers (Roche Applied Science, Indianapolis, IN). Real-time PCR was performed with Power SYBR Green PCR Master Mix in 7900HT Fast Real-Time PCR System (both from Applied Biosystems, Foster City, CA). Amplification of 18S rRNA, hypoxanthine guanine phosphoribosyl transferase (HPRT) and β2-microglobulin (β2MG) were used for sample normalization; data were analyzed using all these endogenous controls and plotted using HPRT only. Primer sequences are as described on Table S2.

RT-PCR data were analyzed by DataAssist software (Applied Biosystems Foster City, CA) using 18S, β2MG and HPRT as endogenous controls, and plotted using HPRT as endogenous control.

### Statistical Analysis

Results are expressed as mean ± standard error. Data were analyzed using unpaired two-tailed student's t test. P values of <0.05 were considered statistically significant.

## Results

### Systemic Anti-Dll4 Treatment Interferes with the BM Vascular Niche

**Figure 1:**
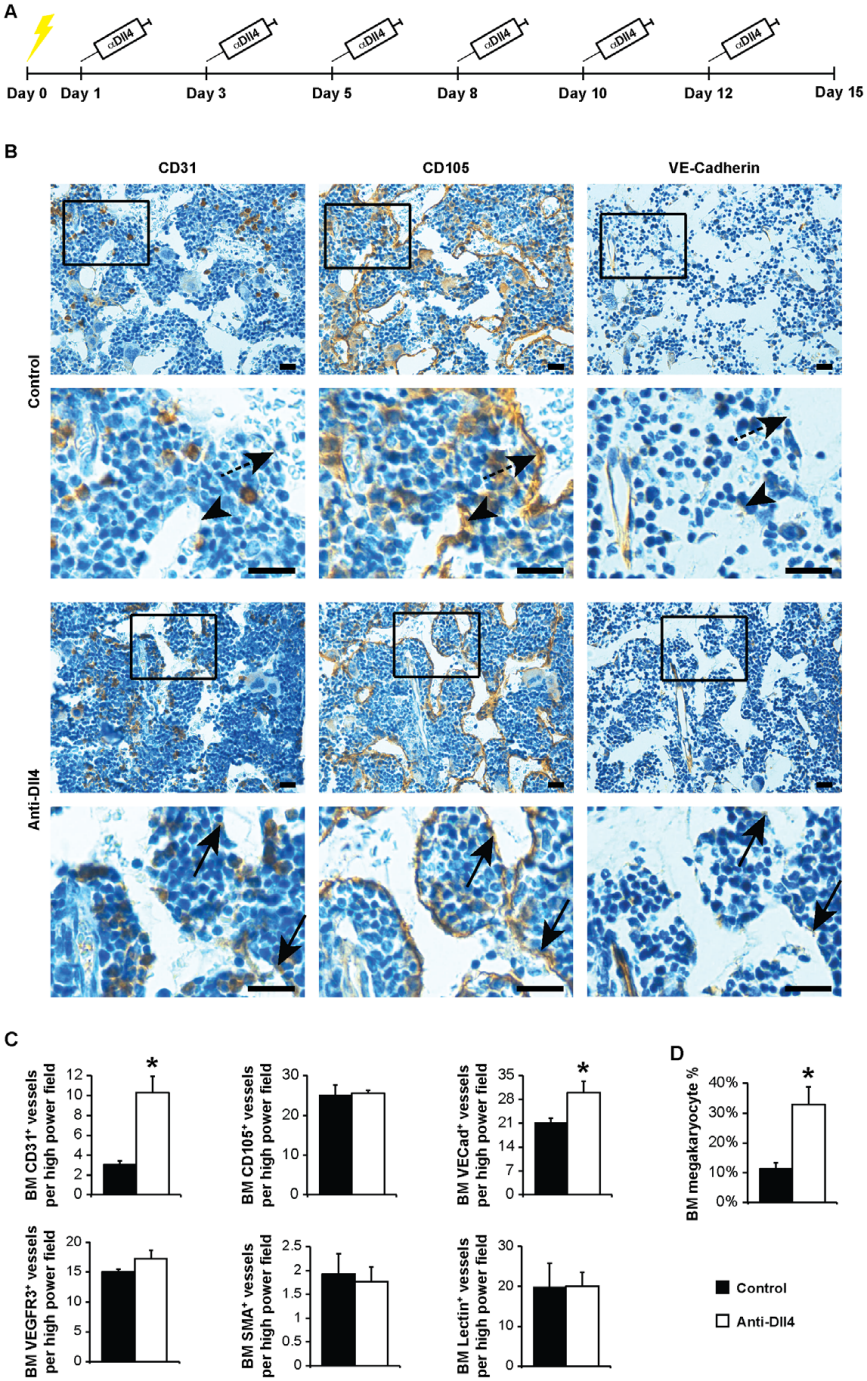
Therapeutic anti-Dll4 blockade interferes with the BM vascular niche. (A) Schematic representation of the clinical assessment of anti-Dll4 treatment. Yellow lightening bolt, sub-lethal irradiation. (B) Immunohistochemistry for CD31, CD105 and VE-Cadherin counterstained with Mayer’s haemalum (Leica DMD 108). Sequential sections represent the same blood vessels. Arrowhead, CD31^-^CD105^+^Ve-Cadherin^+^ blood vessel; dashed arrow, CD31^−^CD105^+^VE-Cadherin^−^ blood vessel; arrow, CD31^+^CD105^+^VE-Cadherin^+^ blood vessel. Bar = 20 µm. (C) CD31, CD105, VE-Cadherin, VEGFR3, SMA and Lectin-positive vessel count, per high power field (400x, Leica DMD 108), reveal an increase of CD31 and VE-Cadherin-positive BM vessels in anti-Dll4 treated mice. (D) Flow cytometric analysis of the percentage of megakaryocytes (CD41^+^ cells) in the BM shows an increase of BM megakaryocyte cell percentage in anti-Dll4 treated mice. Data are means ± s.e.m. *, p<0.05; data represents one of three experiments in which n = 3.

We asked whether a therapeutic (systemic) approach of Dll4 blockade would affect the BM vascular niche. For that, we sub-lethally irradiated mice (300rad), therefore inducing myeloablation and BM turnover, and systemically treated them with a neutralizing anti-Dll4 antibody, HMD4-2 (Figure 1A).

We used six different vascular markers to characterize the effects of anti-Dll4 in the BM vascular niche: CD31, CD105 and VE-Cadherin antibodies, widely used to identify BM endothelial cells [9], [13]; VEGFR3 antibody, described as a specific marker of BM sinusoids [13]; SMA antibody, which labels pericytes in arteries and capillaries [22]-[24]; and *Lycopersicon esculentum* lectin, used as a pan-endothelial marker that stains perfused vessels [18], [54].

By day 15 post-irradiation, increased number of CD31^+^ and VE-Cadherin^+^ vessels were scored in the BM of anti-Dll4 treated mice, with no significant changes in CD105^+^, VEGFR3^+^, SMA^+^, and lectin^+^ vessels (Figure 1B, C, S1A). VE-Cadherin mRNA expression was also increased *in vivo* and *in vitro* following anti-Dll4 treatment (Figure S1B, C).

Furthermore, anti-Dll4 treatment following myeloablation also increased BM megakaryocyte content (Figure 1D). BM VE-Cadherin (endothelial) expression had been previously associated with an increase in megakaryocyte numbers [55]. Moreover, it has been reported that Dll4 impairs the final stages of megakaryocytic differentiation, without affecting its early stages, also concordant with our data [34]. Therefore, the increase in megakaryocyte numbers herein described might be due to the increase in VE-Cadherin expression, to a direct effect of anti-Dll4 treatment on megakaryocytes, or both.

These results were surprising, as previous work has shown quantitative vascular changes in tumors upon anti-Dll4 treatment [17], [18]. However, in our study, we observed a qualitative modulation of the BM vascular niche (as suggested by the use of the different vascular markers). Therefore, we further characterized the type of blood vessels in the BM microenvironment, following anti-Dll4 treatment. As previously described, we found VEGFR3 to be a specific sinusoidal marker [13], lectin to stain all types of blood vessels in the BM [54] (Figure S1A), and SMA to stain for pericyte-covered (stable) blood vessels, such as arteries and capillaries [23], [24] (Figure S1A, S2a,c). CD31, CD105 and VE-Cadherin have been extensively used as BM endothelial cells markers [5], [6], [9], [13], but the CD31 and VE-Cadherin specific modulation led us to further characterize these vessels. As shown in Figure S2, BM stable vessels are CD105^high/low^, VE-Cadherin^high^ and CD31^+^, whereas BM sinusoids are CD105^+^, VE-Cadherin^+/−^, and CD31^+/−^ in sub-lethally irradiated mice.

Next, we asked whether these BM vascular niche-specific changes were a direct effect of Dll4 blockade on the endothelial cells. For that, we used inducible, conditional knockout (VECad-Cre-ER^T2^Dll4^lox/lox^) mice and assessed the number of CD31, CD105 and VE-Cadherin vessels, as well as the percentage of megakaryocytes in the BM. Consistent with the effects reported earlier (seen after systemic anti-Dll4 treatment), we observed a similar phenotype in this genetic targeting of Dll4, with an increase in CD31^+^ and VE-Cadherin^+^ vessels without modulation of CD105^+^ vessels, and an increase in the percentage of CD41^+^ megakaryocytes (Figure S3). These data suggest the effects of anti-Dll4 blockade in the BM vascular niche are exerted predominantly on VE-Cadherin-expressing BM endothelial cells.

The BM vascular modifications herein described were accompanied by systemic defects in the vascular compartment of the liver (Figure S4), as previously reported by others [56].

Together, these data suggest systemic Dll4 blockade perturbs the BM vascular niche, favoring CD31^+^ and VE-Cadherin^+^ endothelial cells expansion and increasing BM megakaryocyte content.

### Specific Effects of Anti-Dll4 Treatment on Endothelial Cells

Next, we investigated the mechanisms by which anti-Dll4 could affect endothelial cells function.

**Figure 2:**
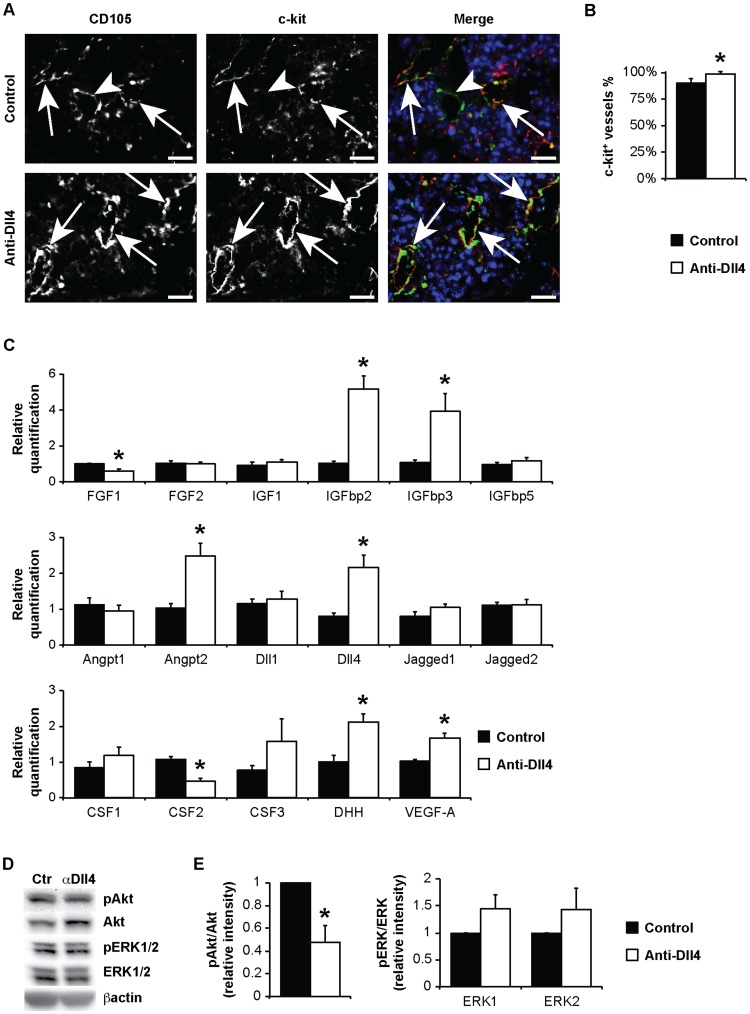
Endothelial-specific effects of anti-Dll4 treatment. (A) Immunofluorescence for CD105 and c-kit (Zeiss AxioImager.Z1). Arrowhead, CD105^+^c-kit^−^ blood vessel; arrow, CD105^+^c-kit^+^ blood vessel. Bar = 20 µm. (B) c-kit^+^(CD105^+^) vessel percentage, per high power field (200x, Zeiss AxioImager.Z1), reveal an increase of c-kit^+^ BM vessel percentage in anti-Dll4 treated mice. (C) Angiocrine gene modulation was accessed by relative quantification of mRNA from total BM, revealing a decrease in FGF1 and CSF2 and an increase of IGFbp2, IGFbp3, Angpt2, Dll4, DHH and VEGF-A expression in anti-Dll4 treated mice. (D) HUVEC phosphorylation of Akt and ERK1/2, analysed by Western blotting. (E) Quantification of phosphorylation of Akt and ERK1/2 relative band intensity reveals a significant increase of Akt phosphorylation. Data are means ± s.e.m. *, p<0.05; n = 3.

First, we characterized the BM endothelial phenotype induced by systemic anti-Dll4 blockade in more detail. We used a stem cell marker, c-kit, and found some BM vessels to be c-kit^+^ (Figure 2A). C-kit is unappreciated as a BM vessel marker, despite *in vitro* reports of c-kit expression in primary BM endothelial cells [57]. Some BM vessels were previously shown to express another stem cell marker, stem cell antigen-1 (Sca-1) [13], but its endothelial functions are still unknown. The overall percentage of c-kit^+^ vessels (assessed from double labeling with CD105) also increased in anti-Dll4 treated animals (Figure 2A, B).

Next, we searched for modulation of “angiocrine genes” and of MAPK and Akt signaling pathways in our system, since these were considered crucial for the instructive role exerted by the BM vascular niche in promoting hematopoietic recovery [29]. We performed qPCR analysis on a set of angiocrine genes, chosen because these are expressed depending on the activation state of BM endothelial cells [29] and because of their involvement in hematopoietic recovery and vascular remodeling (Figure 2C, S5).

Anti-Dll4 treated animals showed a significant decrease in BM expression of fibroblast growth factor 1 (FGF1) and colony stimulating factor 2 (granulocyte-macrophage, CSF2) and an increase in insulin-like growth factor binding protein 2 (IGFbp2), IGFbp3, angiopoietin 2 (Angpt2), Dll4, desert hedgehog (DHH) and vascular endothelial growth factor A (VEGF-A) (Figure 2C, S5A).

This increase in VEGF-A (but not SDF-1α or stem cell factor, SCF) mRNA levels was accompanied by increased VEGF-A protein levels in BM plasma, assessed by ELISA (Figure S5B).

In order to identify endothelial-specific angiocrine gene modulation, we treated HUVEC *in vitro* with anti-Dll4 antibody. Anti-Dll4 treatment resulted in a significant decrease in FGF1, CSF3, but not CSF2, and an increase in VEGF-A expression (Figure S5C). Genes whose expression was not changed *in vivo* were modulated *in vitro*, namely FGF2, CSF3, interleukin 6 (IL-6) and SCF (Figure S5C). Dll4 expression, however, was decreased *in vitro*, and increased *in vivo* (Figure S5C). The latter phenotypes can be interpreted as a non-endothelial cell-specific angiocrine gene modulation; another possibility is that the timing, activation state or endothelial cell identity of this *in vitro* assessment does not mimic BM endothelial cell characteristics.

After characterizing angiocrine gene modulation, we searched for alterations of Akt and ERK1/2 signaling pathways induced by anti-Dll4 treatment. In light of the theory supported by Kobayashi *et al.*, the fine-tuning between Akt and MAPK activation in BM endothelial cells balances self-renewal vs. differentiation of HSPCs. We found that treatment of HUVEC with anti-Dll4 decreased Akt phosphorylation, but did not induce significant changes in ERK1/2 activation (Figure 2D, E), which supports the notion that reduced Akt and equal MAPK promotes the maintenance of the HSPCs pool [29].

These data suggest that modulating the BM vascular niche by anti-Dll4 treatment increases c-kit^+^ vessels and affects BM endothelial cells activation state and angiocrine factors production.

### Anti-Dll4 Treatment Perturbs Hematopoietic Recovery Following Irradiation

Having shown systemic anti-Dll4 treatment affected BM endothelial cells *in vivo* and *in vitro*, including angiocrine gene modulation, next we explored the hematopoietic effects of anti-Dll4 treatment in BM hematopoietic recovery following myeloablation.

**Figure 3:**
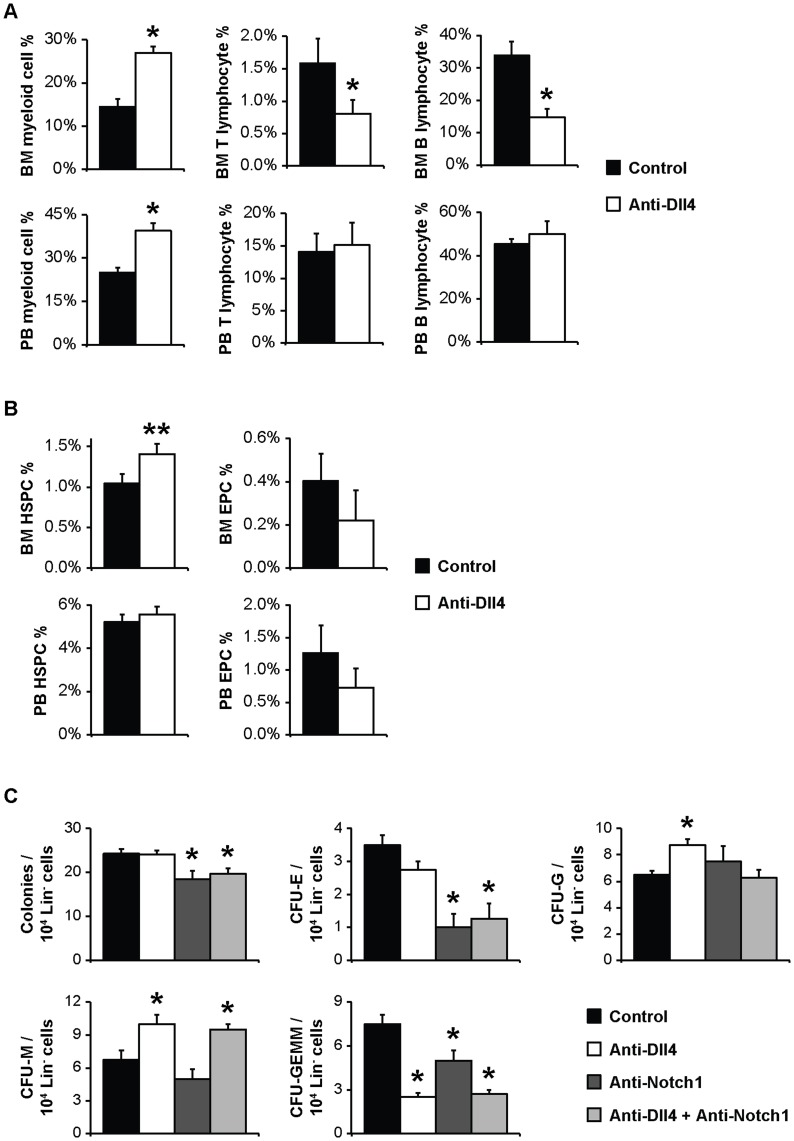
Anti-Dll4 treatment perturbs hematopoiesis following irradiation. (A) Flow cytometric analysis of the percentage of myeloid (CD11b^+^) cells, T lymphocytes (CD3^+^ cells), and B lymphocytes (B220^+^) in the BM and PB, revealing an increase in both myeloid BM and PB content and a decrease in T and B lymphocyte BM content in anti-Dll4 treated mice. (B) Flow cytometric analysis of the percentage of stem/progenitor cells, namely HSPCs (Sca1^+^Flk1^−^) and EPCs (Sca1^+^Flk1^+^), revealing that anti-Dll4 treatment does not significantly affect these populations, notwithstanding the trend (p = 0.07) towards and increase of BM HSPCs. Data are means ± s.e.m. *, p<0.05, **, p = 0.07; data represents one of three experiments in which n = 3. (C) Colony counts from methylcellulose culture of Lin^-^ cord blood-derived cells reveal anti-Dll4 treatment *in vitro* induces an increased HSPCs potential to differentiate to the myeloid lineage (CFU-G and CFU-M), an effect independent upon anti-Notch1 treatment. Anti-Notch1 treatment, independent of combined anti-Dll4 treatment, induces a decrease in HSPCs potential to differentiate to the erythrocytic lineage (CFU-E), and decreased HSPCs differentiation potential (total colony number). All treatments reduced multipotent HSPCs (CFU-GEMM). Data are means ± s.e.m. *, p<0.05; n = 4.

Both BM and PB from anti-Dll4 treated mice showed increased myeloid cell content (CD11b^+^) (Figure 3A). The BM lymphocytic compartment was also affected by the anti-Dll4 treatment; there was a significant decrease in both CD3^+^ T and B220^+^ B lymphocytes, with no significant changes in the PB (Figure 3A).

In contrast, anti-Dll4 treatment did not seem to affect BM progenitor cell populations. As shown in Figure 3B, there were no significant changes in the percentage of BM or PB EPCs (Sca1^+^Flk1^+^) or HSPCs (Sca1^+^Flk1^−^), with a trend for increased BM HSPCs (p = 0.07) in anti-Dll4 treated mice.

After characterizing the global alterations in hematopoiesis upon anti-Dll4 treatment, we performed *in vitro* CFU assays, counting single-cell derived colonies, which represent either multipotent (CFU-granulocyte-erythrocyte-macrophage-megakaryocyte, CFU-GEMM), bipotent (CFU-granulocyte-macrophage) or unipotent (CFU-monocyte, CGU-M, CFU-granulocyte, CFU-G, or CFU-erythrocyte, CFU-E) [47], [48]. These assays allowed us to evaluate if the hematopoietic effects seen with anti-Dll4 treatment could also be due to direct effects on hematopoietic elements, namely in their differentiation capacity.

For that, we sorted BM HSPCs (Lin^-^Sca1^+^) from anti-Dll4 treated and control mice and cultured these in methylcellulose *in vitro* [47], [48]. In accordance with the lack of change in HSPCs frequency seen after anti-Dll4 treatment (Figure 3B), this did not affect HSPCs CFU potential, or colony number (Figure S6).

Next, we also assessed the direct effects of anti-Dll4 treatment on HSPCs, by treating naïve HSPCs with anti-Dll4 *in vitro*, in CFU assays. We further sought to determine whether Notch1 was the receptor involved, by blocking Notch1 using a monoclonal antibody either alone or in conjugation with anti-Dll4. We induced cord blood HSPCs’ (Lin^-^) differentiation in methylcellulose in the presence of either PBS, anti-Dll4, anti-Notch1, or the 2 neutralizing antibodies together. As shown in Figure 3C, anti-Dll4 treatment shifted differentiation towards the myeloid lineage (increased CFU-M and CFU-G colonies), an effect independent of anti-Notch1 treatment, as anti-Notch1 did not affect CFU-M or CFU-G colony number. Anti-Dll4 treatment reduced multipotent HSPCs (CFU-GEMM colonies), as did anti-Notch1 and the conjugation of both antibodies, indicating that anti-Dll4 treatment reduced multipotent HSPCs by reducing Notch1-mediated Notch signalling. Anti-Notch1, alone or combined with anti-Dll4, decreased HSPCs potential to differentiate into the erythroid lineage (CFU-E), and decreased HSPCs differentiation potential (total colony number). Both treatments reduced multipotent HSPCs (CFU-GEMM) (Figure 3C).

Taken together, these data suggest that besides affecting the BM vascular niche, anti-Dll4 treatment also perturbs hematopoietic cell differentiation and commitment.

### Anti-Dll4 Treatment of Donor BM Improves Hematopoietic Recovery Following Transplantation into Lethally Irradiated Recipients

**Figure 4:**
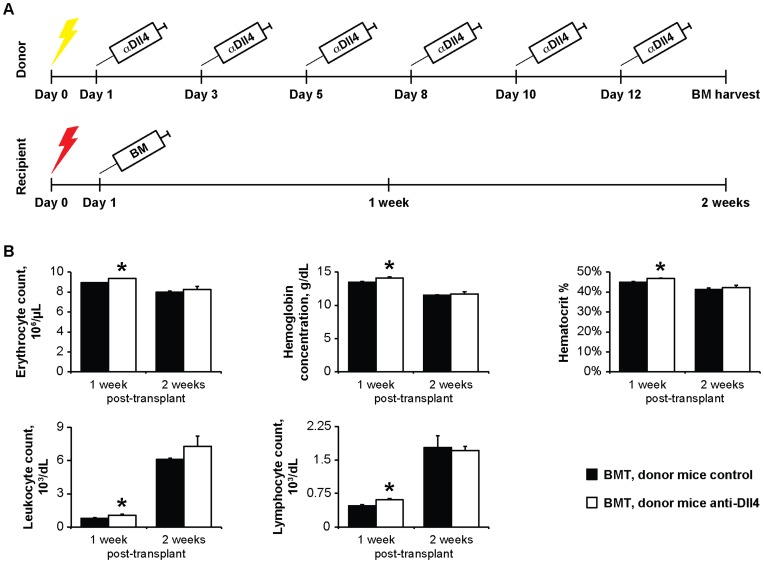
Anti-Dll4 treatment of donor BM improves hematopoietic recovery following transplantation into lethally irradiated recipients. (A) Schematic representation of the BMT. Yellow lightening bolt, sub-lethal irradiation; red lightening bolt, lethal irradiation. (B) Erythrocyte, hemoglobin, hematocrit, leukocyte and lymphocyte quantifications were assessed by PB cell blood counts. Data shows donor anti-Dll4 treated mice induces faster recovery of different hematological parameters day 1 week after transplantation. Data are means ± s.e.m. *, p<0.05; n = 3.

Next, we assessed whether the BM changes induced by anti-Dll4 treatment affected the efficiency of BM hematopoietic recovery in a transplant setting. For this purpose, we lethally irradiated recipient mice, which were subsequently transplanted with BM from untreated or anti-Dll4 treated mice (Figure 4A). Mice that received BM from anti-Dll4 treated mice showed evidence of improved hematopoietic recovery following lethal myeloablation (significantly faster recovery of leukocytes, hematocrit and lymphocytes), assessed by CBC (Figure 4B).

These data suggest that treatment of BM donor mice with anti-Dll4 improves hematopoietic recovery following lethal myeloablation.

## Discussion

The data presented in this paper show that systemic Dll4 blockade induces qualitative changes in the BM vasculature (which becomes more heterogeneous), which may be favorable in a BMT setting. A number of studies have proven the relevance of the BM vascular niche in hematopoiesis, but the heterogeneity of the BM vasculature has only been recently objectively assessed, clearly suggesting further detailed studies are needed to understand the importance of the different BM vessels for normal BM function [4]–[9], [13], [26], [29], [58]–[60].

We describe that systemic targeting Dll4, which has previously been shown to confine to particular vascular ECs (called “tip cells”), changes the vascular identity in the BM. Following myeloablation, we applied an anti-Dll4 treatment, similar to what is currently being performed in phase I clinical trials to treat patients with solid malignancies. This treatment resulted in different vascular alterations in the BM, as shown by increased CD31, VE-Cadherin and c-kit^+^ cells, without quantitative changes in CD105^+^, VEGFR3^+^, SMA^+^ or lectin^+^ vessels. The global BM vessel identity is therefore altered upon anti-Dll4 treatment.

Interestingly, CD31 is indispensable for several stages of hematopoiesis, endothelial cells survival and angiogenesis, which in turn are all crucial for hematopoietic recovery following myeloablation [13], [26], [61]–[67]. VE-Cadherin is also required for hematopoiesis and angiogenesis [55], [68], [69]. The role of c-kit, in ECs, however, is unknown; studies assessing its role in angiogenesis and, more specifically, in the BM microenvironment, will be required for proper interpretation of the data presented in this paper.

Regarding the modulation of “angiocrine genes”, besides the increase in CD31^+^ BM vessels previously described, we detected a significant increase in IGFbp2, IGFbp3, Angpt2, DHH and VEGF-A and a decrease in FGF1 and CSF2 expression in whole BM extracts from anti-Dll4 treated animals (Figure 2C). Even though FGF1, which is decreased upon anti-Dll4 treatment, prevents vessel regression, IGFbp3, Angpt2 and VEGF-A, which are increased, are modulators of vascular survival and re-growth, which, as previously mentioned, is crucial for hematopoietic recovery following myeloablation [70]–[72].

Despite the decrease of CSF2, which is associated with a decrease in the myeloid lineage, IGF1 induces proliferation and differentiation of myeloid lineage cells [73], and DHH is important for granulocyte differentiation/proliferation in the BM [74]; moreover, the myeloid modulation we describe may be due to a direct effect of anti-Dll4 treatment on hematopoietic cells (Figure 3C). Both HSPCs and myeloid cells are reported to express Dll4 [32]. We show that anti-Dll4 treatment of HSPCs *in vitro* increases CFU-M and CFU-G number, independently of Notch1 modulation, but decreases multipotential progenitor cell-derived CFUs, similar to anti-Notch1 treatment (Figure 3C).

VEGF-A blocks both B and T lymphopoiesis [75]–[77]. The altered BM lymphocyte content observed might either be simply due to the increased myeloid content, to the VEGF-A increase in the BM, to the direct effect of anti-Dll4 in lymphoid cells, and/or to the effect of Dll4 inhibition in secondary hematopoietic organs, such as the thymus and spleen, which were previously shown to express Dll4 [35], [49], [56], [78]–[80].

Regarding the signaling pathways that are proposed to trigger the EC role in hematopoiesis, we observed a decrease of Akt activation, without significant changes in Erk1/2 (Figure 2D, E) after exposing EC to anti-Dll4. It should be noted that Dll4 has been shown to modulate the MAPK activation on a stimulus-depending manner [81]; the technical constraints to study EC-specific signaling pathways activation *in vivo* led us to an *in vitro* study, which may not completely mirror the *in vivo* systemic effects of anti-Dll4, nor the proper stimulus acting in different BM microenvironments.

*In vivo* assessments showed both HSPC phenotype and function (reconstitution potential) were not impaired by systemic anti-Dll4 treatment, and *in vitro* differentiation of HSPCs collected from the BM of anti-Dll4 treated mice was also not impaired, meaning HSPCs are unaffected by *in vivo* anti-Dll4 treatment (Figure 3B, 4B and S6).

Systemic anti-Dll4 treatment of donor mice in a setting of BMT resulted in a mild, but significant, accelerated hematopoietic recovery of recipient mice (Figure 4) [7], [13]. For a successful BMT, HSPCs must home and engraft in the BM, a process for which BM ECs are essential [11]–[13]. In this study, we transplanted whole BM mononuclear cells; this fraction includes BM ECs, which were previously shown to incorporate in the BM vasculature [12]. Interestingly, vascular CD31 and VE-Cadherin regulate the transition of HSPCs between blood and BM [65], [68]. The increased VE-Cadherin and CD31-positive BM vessels from anti-Dll4 treated donor mice may have enhanced the homing of HSPCs in recipient mice, thereby leading to an overall faster hematopoietic recovery. Interestingly, we have also observed an increase in Dll4 expression in the BM of anti-Dll4 treated donor mice (Figure 2C). Given that anti-Dll4 treatment was performed only in donor mice, and not in recipients, the transplanted cells may have increased Dll4 protein levels. Remarkably, *in vitro* data have shown that increasing Dll4 signaling in HSPCs increases erythroid commitment and HSPCs proliferation, induces commitment and complete maturation to the T cell lineage, and maintains HSPCs stemness [32], [34], [82], [83]. In our BMT model, these effects were transient, because the treatment was not maintained thoughout the process of hematopoietic recovery; therefore, the lymphoproliferative disease that mice overexpressing Dll4 in the hematopoietic lineage are expected to develop was not observed (evidenced by the long term survival of recipient mice) [33], [35]. Alternatively, or in addition, IGFbp2 and IGFbp3 showed increased expression following anti-Dll4 treatment; these factors, by stabilizing IGF1, may contribute towards the effects of anti-Dll4 in promoting hematopoietic recovery following BMT [84].

Together, our data shows that targeting Dll4 alters the vascular identity in the BM, mildly affects hematopoiesis, and promotes a faster hematopoietic recovery after BMT. We have characterized the BM vascular niche and provide evidence of its heterogeneity, which may create different microenvironments within the BM. This assessment may be particularly interesting to explore, as relevant information regarding the functional characterization of hematopoietic stem cell niches can be obtained. We further suggest anti-Dll4 blockade may be an interesting therapeutic approach in a BMT setting.

## Supporting Information

Figure S1 **Anti-Dll4 blockade interferes with the BM vascular niche.** (A) Immunohistochemistry for VEGFR3 and SMA counterstained with Mayer’s haemalum (Leica DMD 108). Immunofluorescence for lectin (Leica LSM 510). Bar = 20 µm. (B) Relative quantification of mRNA from total BM reveals an increase in VE-Cadherin, but not CD31, expression in anti-Dll4 treated mice. (C) Relative quantification of mRNA from HUVEC reveals an increase in VE-Cadherin, but not CD31, expression in anti-Dll4 treated cells. Data are means ± s.e.m. *, p<0.05; n = 3.
(TIF)

Figure S2 **The use of different endothelial cell markers reveal different types of BM vessels.** Immunohistochemistry for CD105, VE-Cadherin, SMA and CD31 counterstained with Mayer’s haemalum (LEICA DMD 108). Stable vessels are SMA^+^ (a, c), CD105^high^ (a) or CD105^low^ (c, f), VE-Cadherin^high^ (a, c, f), and CD31^+^ (f). Sinusoids are SMA^-^ (b, d, e), CD105^+^ (b, d, e, g, h), VE-Cadherin^+^ (b, e) or VE-Cadherin^-^ (d), and CD31^+^ (g) or CD31^-^ (h). Bar = 10 µm.
(TIF)

Figure S3 **Endothelial cell-specific Dll4 blockade interferes with the BM vascular niche.** (A) Immunohistochemistry for CD31, CD105 and VE-Cadherin counterstained with Mayer’s haemalum (Leica DMD108). Bar = 20 µm. (B) CD31, CD105 and VE-Cadherin-positive vessel count, per high power field (400x, Leica DMD108), reveal an increase of CD31 and VE-Cadherin-positive BM vessels in VECad-Cre-ER^T2^Dll4^lox/lox^ mice. (C) Flow cytometry analysis of the percentage of megakaryocytes (CD41^+^ cells) in the BM shows an increase of BM megakaryocyte cell percentage in mice. Data are shown as means ± s.e.m. *, p<0.05; n = 11.
(TIF)

Figure S4 **Therapeutic anti-Dll4 blockade interferes with the hepatic vascular niche.** (A) Macroscopic observation of the liver of anti-Dll4 treated mice reveals an obvious disruption in tissue architecture. Bar = 2 mm. (B) Histology of the liver reveals anti-Dll4 treatment promotes severe centrolobular sinusoidal dilation (arrows), with multifocal hepatocyte regeneration foci (arrowheads), as compared to the normal liver morphology observed in control mice; hematoxilin-eosin staining (Leica DMD 108). Bar = 25 µm. Data are means ± s.e.m. *, p<0.05; data represents one of three experiments in which n = 3.
(TIF)

Figure S5 **Endothelial-specific effects of anti-Dll4 treatment.** (A) Angiocrine gene modulation was assessed by relative quantification of mRNA from total BM. None of the displayed genes is modulated *in vivo* by anti-Dll4 treatment. (B) Bone marrow VEGF-A, SDF-1α and SCF levels, as determined by ELISA. (C) Angiocrine gene modulation was assessed *in vitro* by relative quantification of mRNA from HUVEC. HUVEC subjected to anti-Dll4 treatment decreases FGF1 and increases VEGF-A expression, similar to total BM from anti-Dll4 treated mice. CSF3, but not CSF2, expression is decreased upon *in vitro* anti-Dll4 treatment. FGF2 and Dll4 are significantly decreased, and IL-6 and SCF are significantly increased in anti-Dll4 treated cells. Data are means ± s.e.m. *, p<0.05; n = 3.
(TIF)

Figure S6 **Anti-Dll4 treatment does not perturb colony forming (CFU) potential of Lin-Sca1+ hematopoietic precursor cells.** Colony counts from methylcellulose culture of Lin^-^Sca1^+^ sorted cells reveal anti-Dll4 treatment *in vivo* does not affect intrinsic stem cell’s ability to differentiate into different hematopoietic lineages. Data are means ± s.e.m. *, p<0.05; n = 3.
(TIF)

Table S1 **Antibodies list.**
(XLS)

Table S2 **Primers list.**
(XLS)
