# Supplementary figures and images for: Context- and Cell-Dependent Effects of Delta-Like 4 Targeting in the Bone Marrow Microenvironment
Source: PLoS One. 2012 Dec 20;7(12):e52450. doi: 10.1371/journal.pone.0052450 (PMC3527506; doi:10.1371/journal.pone.0052450)

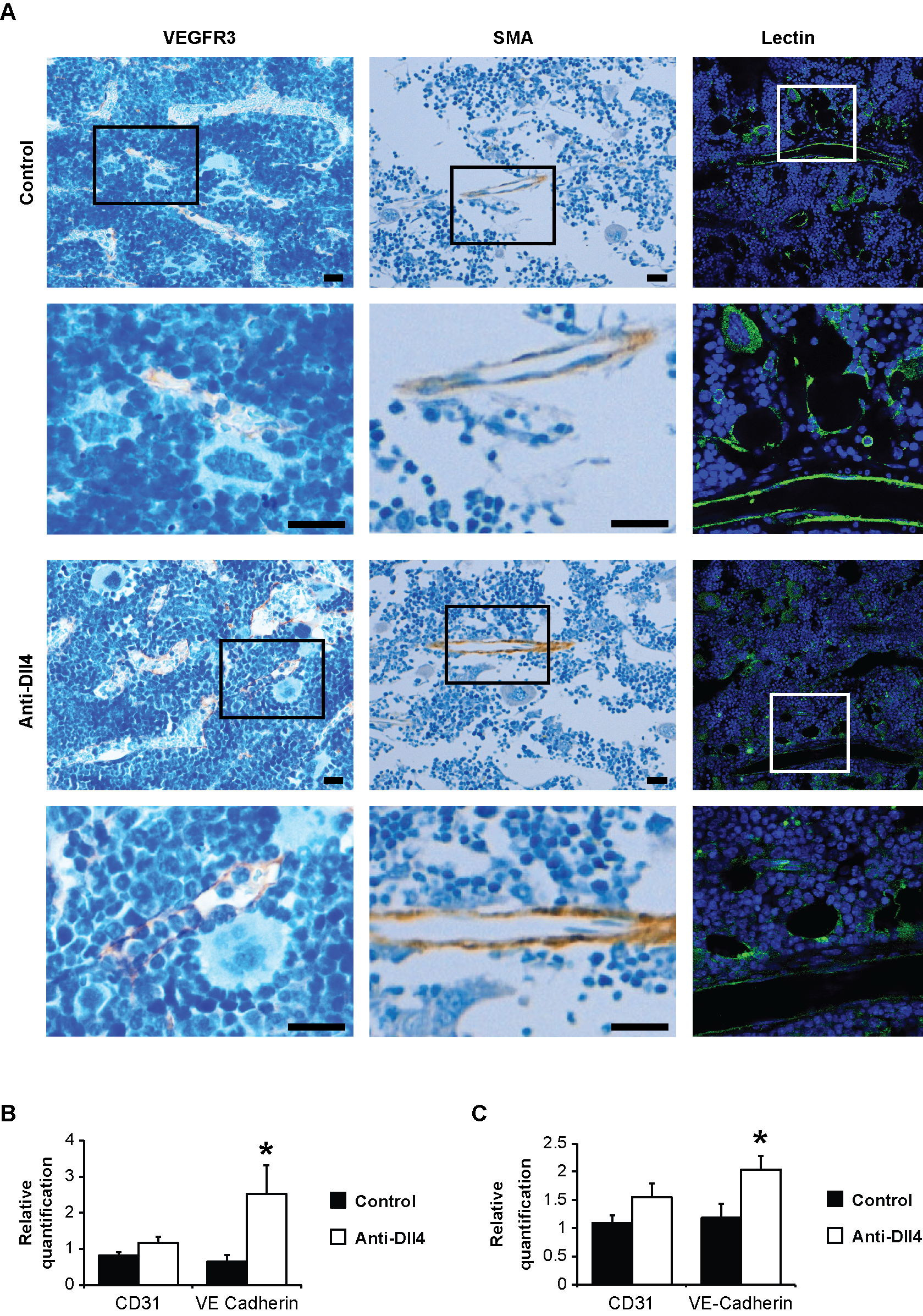

Supplement: Figure S1 — Anti-Dll4 blockade interferes with the BM vascular niche. (A) Immunohistochemistry for VEGFR3 and SMA counterstained with Mayer’s haemalum (Leica DMD 108). Immunofluorescence for lectin (Leica LSM 510). Bar = 20 µm. (B) Relative quantification of mRNA from total BM reveals an increase in VE-Cadherin, but not CD31, expression in anti-Dll4 treated mice. (C) Relative quantification of mRNA from HUVEC reveals an increase in VE-Cadherin, but not CD31, expression in anti-Dll4 treated cells. Data are means ± s.e.m. *, p<0.05; n = 3. (TIF) [file pone.0052450.s001.tif]

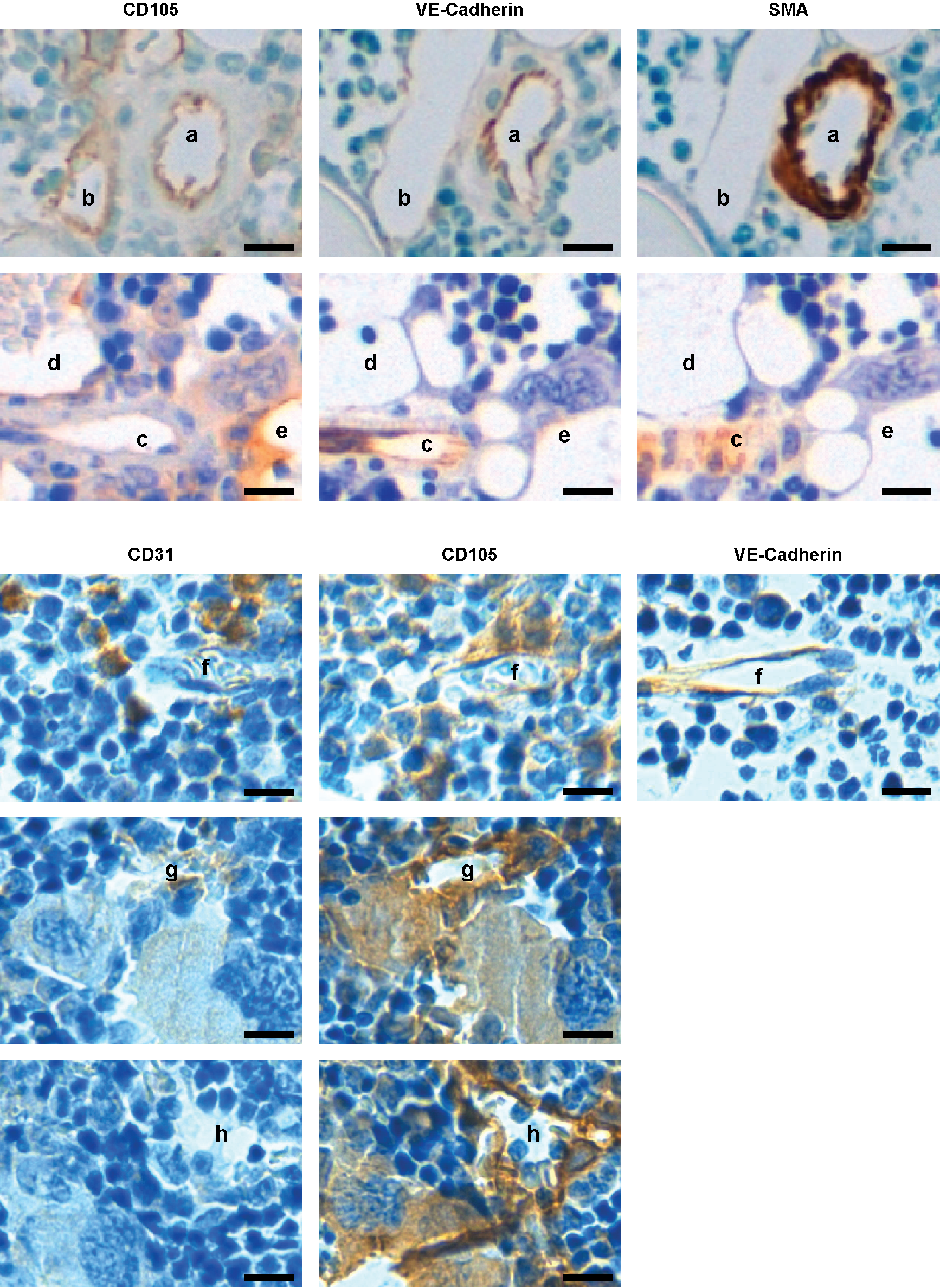

Supplement: Figure S2 — The use of different endothelial cell markers reveal different types of BM vessels. Immunohistochemistry for CD105, VE-Cadherin, SMA and CD31 counterstained with Mayer’s haemalum (LEICA DMD 108). Stable vessels are SMA+ (a, c), CD105high (a) or CD105low (c, f), VE-Cadherinhigh (a, c, f), and CD31+ (f). Sinusoids are SMA- (b, d, e), CD105+ (b, d, e, g, h), VE-Cadherin+ (b, e) or VE-Cadherin- (d), and CD31+ (g) or CD31- (h). Bar = 10 µm. (TIF) [file pone.0052450.s002.tif]

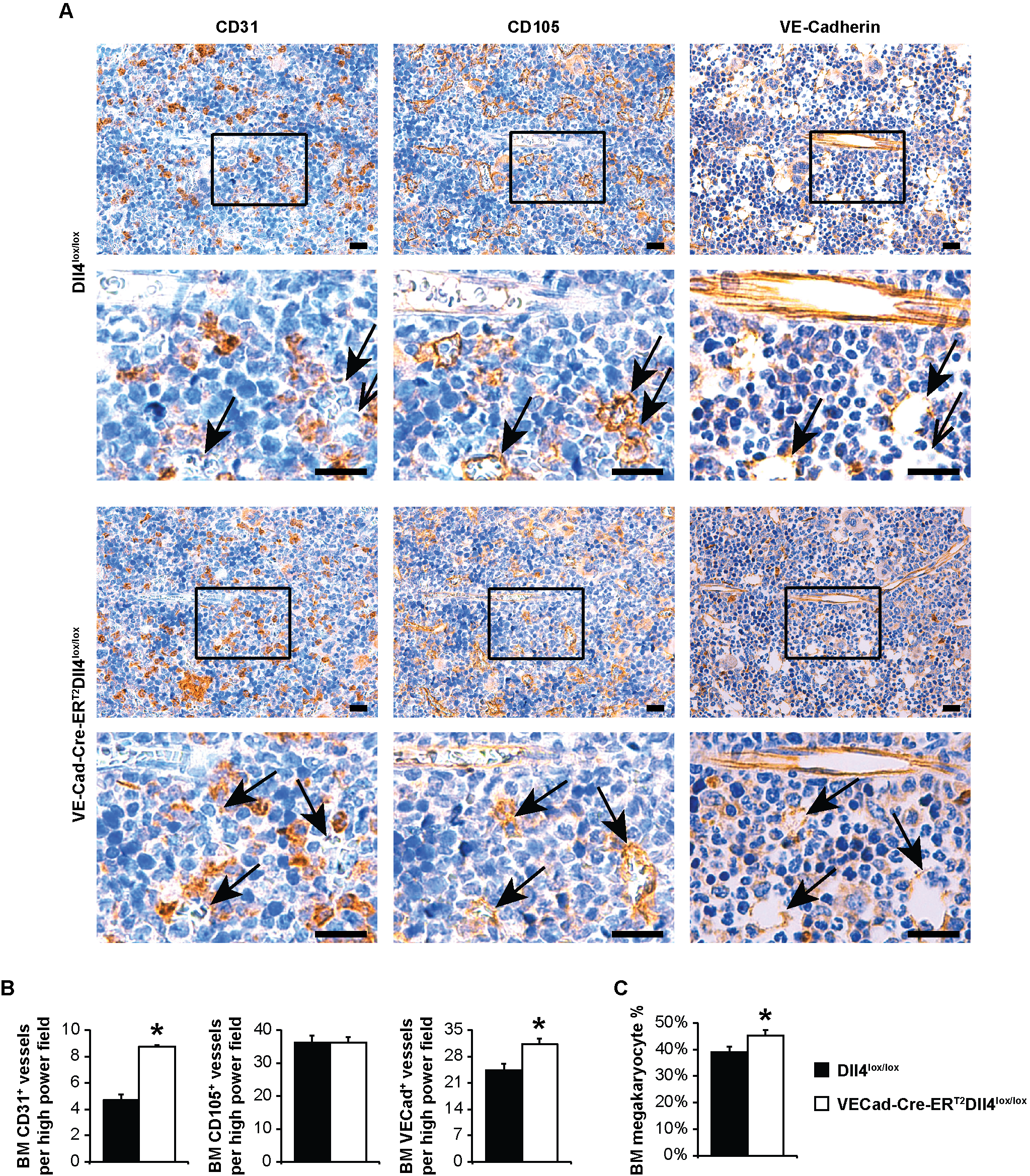

Supplement: Figure S3 — Endothelial cell-specific Dll4 blockade interferes with the BM vascular niche. (A) Immunohistochemistry for CD31, CD105 and VE-Cadherin counterstained with Mayer’s haemalum (Leica DMD108). Bar = 20 µm. (B) CD31, CD105 and VE-Cadherin-positive vessel count, per high power field (400x, Leica DMD108), reveal an increase of CD31 and VE-Cadherin-positive BM vessels in VECad-Cre-ERT2Dll4lox/lox mice. (C) Flow cytometry analysis of the percentage of megakaryocytes (CD41+ cells) in the BM shows an increase of BM megakaryocyte cell percentage in mice. Data are shown as means ± s.e.m. *, p<0.05; n = 11. (TIF) [file pone.0052450.s003.tif]

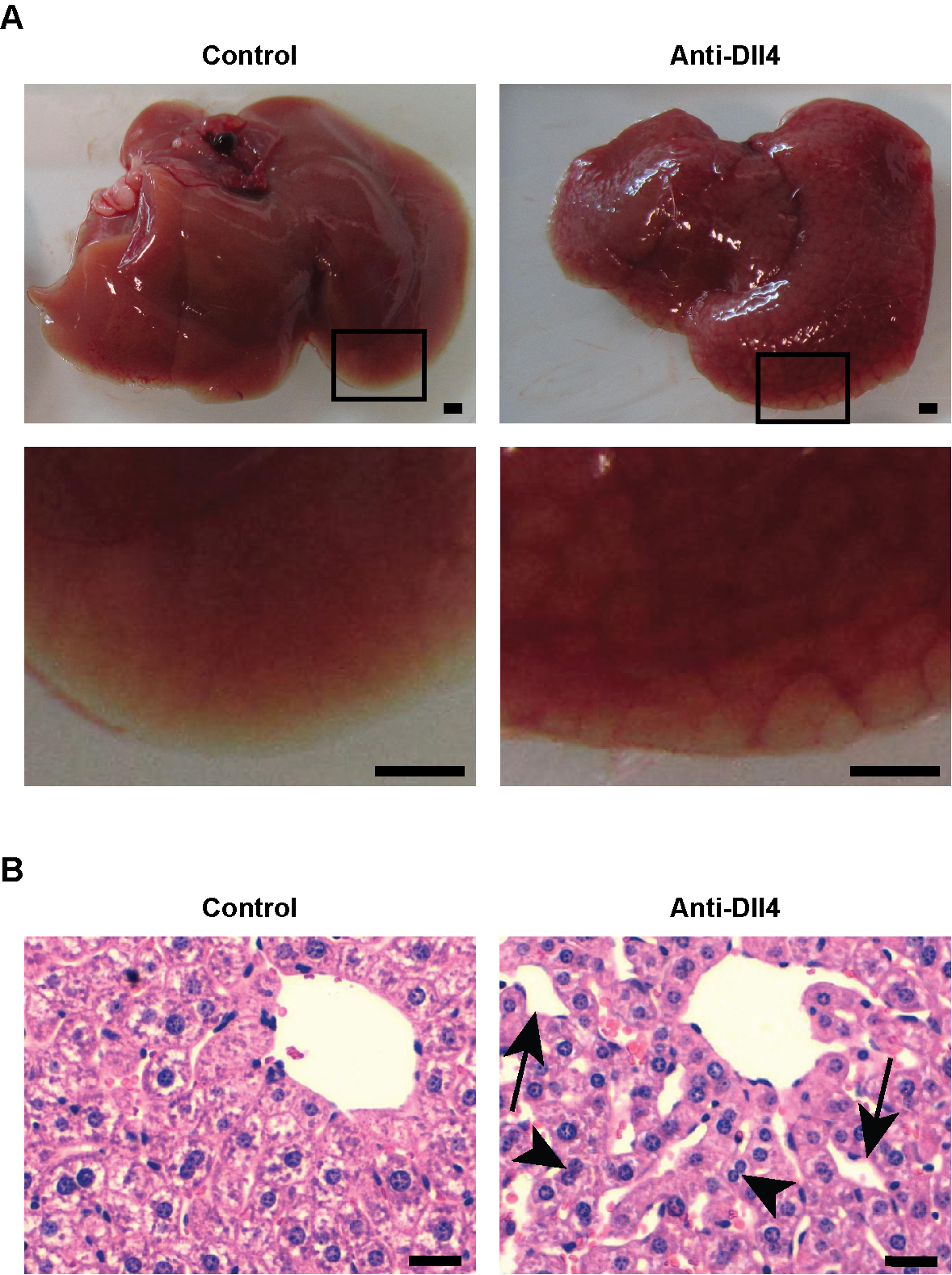

Supplement: Figure S4 — Therapeutic anti-Dll4 blockade interferes with the hepatic vascular niche. (A) Macroscopic observation of the liver of anti-Dll4 treated mice reveals an obvious disruption in tissue architecture. Bar = 2 mm. (B) Histology of the liver reveals anti-Dll4 treatment promotes severe centrolobular sinusoidal dilation (arrows), with multifocal hepatocyte regeneration foci (arrowheads), as compared to the normal liver morphology observed in control mice; hematoxilin-eosin staining (Leica DMD 108). Bar = 25 µm. Data are means ± s.e.m. *, p<0.05; data represents one of three experiments in which n = 3. (TIF) [file pone.0052450.s004.tif]

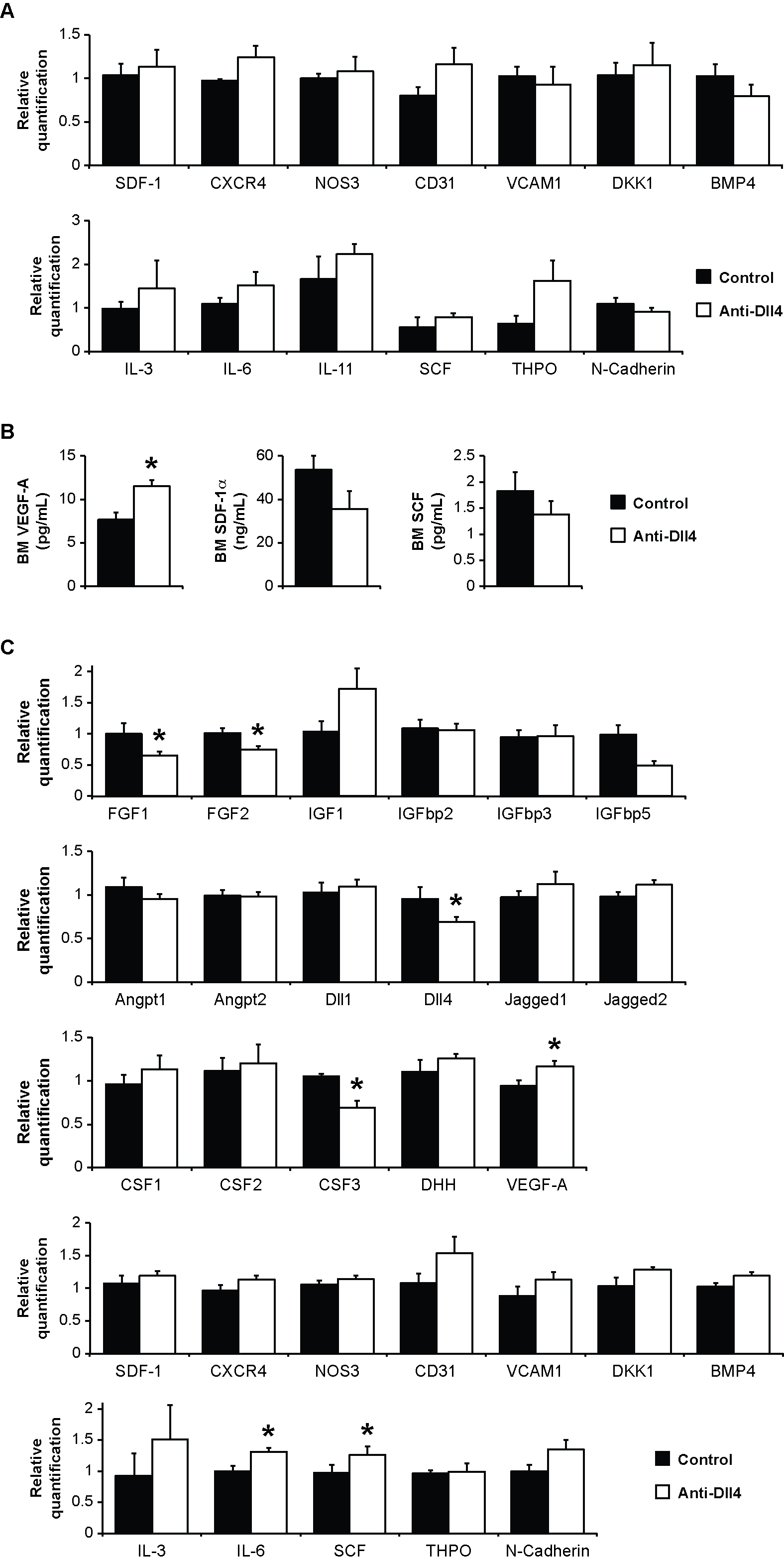

Supplement: Figure S5 — Endothelial-specific effects of anti-Dll4 treatment. (A) Angiocrine gene modulation was assessed by relative quantification of mRNA from total BM. None of the displayed genes is modulated in vivo by anti-Dll4 treatment. (B) Bone marrow VEGF-A, SDF-1α and SCF levels, as determined by ELISA. (C) Angiocrine gene modulation was assessed in vitro by relative quantification of mRNA from HUVEC. HUVEC subjected to anti-Dll4 treatment decreases FGF1 and increases VEGF-A expression, similar to total BM from anti-Dll4 treated mice. CSF3, but not CSF2, expression is decreased upon in vitro anti-Dll4 treatment. FGF2 and Dll4 are significantly decreased, and IL-6 and SCF are significantly increased in anti-Dll4 treated cells. Data are means ± s.e.m. *, p<0.05; n = 3. (TIF) [file pone.0052450.s005.tif]

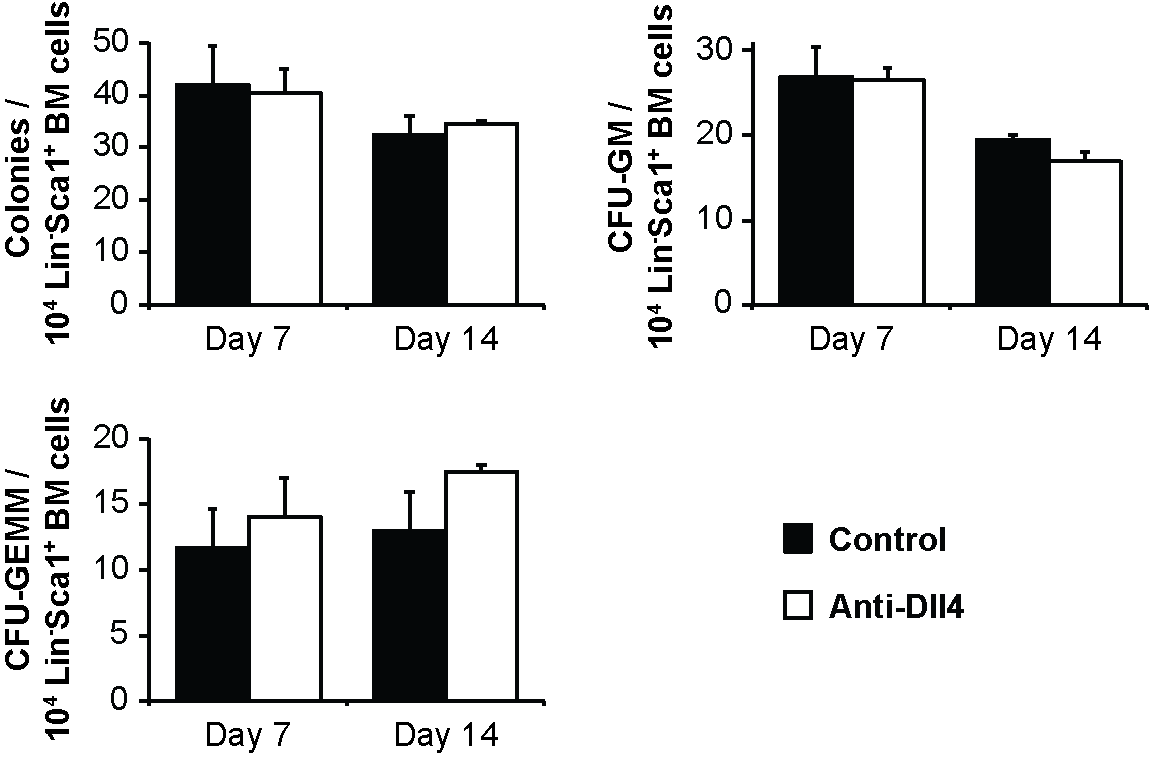

Supplement: Figure S6 — Anti-Dll4 treatment does not perturb colony forming (CFU) potential of Lin-Sca1+ hematopoietic precursor cells. Colony counts from methylcellulose culture of Lin-Sca1+ sorted cells reveal anti-Dll4 treatment in vivo does not affect intrinsic stem cell’s ability to differentiate into different hematopoietic lineages. Data are means ± s.e.m. *, p<0.05; n = 3. (TIF) [file pone.0052450.s006.tif]
